# Automated microbatch-under-oil phase diagrams to rationalize serial crystallography sample preparation

**DOI:** 10.1107/S2052252526000448

**Published:** 2026-01-27

**Authors:** Jack Stubbs, Courtney J. Tremlett, Abigail Waitman, Nicholas J. Harmer, Allen M. Orville, Ivo Tews, Stefan Kolek, Patrick D. Shaw Stewart

**Affiliations:** a School of Biological Sciences, Faculty of Environmental and Life Sciences University of Southampton SouthamptonSO17 1BJ United Kingdom; b Diamond Light Source Harwell Science and Innovation Campus DidcotOX11 0DE United Kingdom; c Living Systems Institute University of Exeter Stocker Road ExeterEX4 4QD United Kingdom; d Department of Biosciences University of Exeter Stocker Road ExeterEX4 4QD United Kingdom; e Institute for Life Sciences University of Southampton SouthamptonSO17 1BJ United Kingdom; f Research Complex at Harwell Harwell Science and Innovation Campus DidcotOX11 0FA United Kingdom; g Douglas Instruments Ltd, East Garston, HungerfordRG17 7HD, United Kingdom; King’s College London, United Kingdom; University of Padova, Italy

**Keywords:** serial crystallography, sample preparation, phase diagrams, microcrystals, microbatch-under-oil, seeding, optimization

## Abstract

Serial crystallography relies on the reproducible production of high-density suspensions of microcrystals, yet sample optimization remains a resource-intensive bottleneck. While phase diagrams provide a theoretical framework for controlling crystal size and number, experimental mapping is traditionally hindered by relatively high sample consumption. We present an automated microbatch-under-oil crystallization approach that rapidly maps phase boundaries using only 15–60 µl (∼0.15–3.8 mg) of protein. While this workflow is ideally suited for refining existing hits, it serves as a standalone platform for characterizing the crystallization landscape of new protein targets. The power of this approach lies in the integration of three distinct strategies that exploit the stable chemical environment of microbatch-under-oil. Firstly, we utilize an ingenious diagonal sampling strategy that traverses the phase boundary parallel to the solubility curve by systematically varying protein-to-precipitant ratios, identifying primary nucleation zones with far greater efficiency than traditional orthogonal grids. Secondly, we employ a linked variation of multiple precipitants to reveal morphology-specific regions, such as the rod versus plate transitions crucial for time-resolved experiments. Finally, we incorporate automated seed-stock titration to precisely define the metastable zone, enabling the predictive rescue of nucleation-limited systems. The synergy of these three strategies enables the systematic decoupling of nucleation from growth, providing a rational route to optimize microcrystal density, size and lattice order. Crucially, by eliminating the evaporation-related variables inherent in vapor diffusion, this method ensures that the chemical coordinates identified during screening remain constant during scale-up to larger volumes. This workflow transforms empirical serial crystallography sample preparation into a rational, reproducible and highly efficient process applicable to both the optimization of known conditions and the *de novo* development of microcrystal suspensions, tailored to the rigorous demands of modern serial diffraction experiments.

## Abstract 2

An automated, low-volume microbatch-under-oil crystallization approach is described that rapidly maps phase diagram boundaries. This approach rationalizes the production of microcrystal suspensions for serial crystallography by explicitly distinguishing metastable from nucleation zones, thereby replacing empirical trial and error with a quantitative guide to sample optimization.

## 1. Introduction

Serial crystallography (SX) at X-ray free-electron lasers (XFELs) and fourth-generation synchrotrons (Chapman *et al.*, 2011 ▸; Stellato *et al.*, 2014 ▸; Barends *et al.*, 2022 ▸) has fundamentally transformed structural biology (Henkel & Oberthür, 2024 ▸). By merging diffraction data from thousands of microcrystals, SX enables structure determination at physiological temperatures and facilitates time-resolved studies of dynamic processes (Caramello & Royant, 2024 ▸; Banari *et al.*, 2025 ▸). However, the efficacy of SX depends on the availability of sufficient quantities of uniformly sized microcrystals (Manna *et al.*, 2025 ▸; Tremlett *et al.*, 2025 ▸). Consequently, transitioning from single-crystal growth to homogenous microcrystal suspensions remains a primary bottleneck.

Macromolecular crystallization is governed by the phase diagram, mapping protein and precipitant concentrations to distinct regions: undersaturated, metastable (crystal growth) and labile (nucleation and precipitation) (George & Wilson, 1994 ▸; Asherie, 2004 ▸; Rupp, 2015 ▸). While notional phase diagrams guide initial screening (Luft *et al.*, 2011 ▸), actual phase boundaries are often unpredictable (Saridakis *et al.*, 1994 ▸; Chayen, 1999 ▸). Empirical phase diagram determination is therefore critical for defining reproducible conditions for optimal microcrystal growth, as crystal morphology, size distribution and diffraction quality can vary significantly across the phase space (Beale *et al.*, 2019 ▸).

Microbatch-under-oil crystallization offers distinct advantages for phase diagram mapping compared with vapor diffusion. By sealing the mixture under an oil layer (Chayen *et al.*, 1990 ▸, 1992 ▸; Blow *et al.*, 1994 ▸), solvent evaporation is effectively eliminated (Chayen, 1997 ▸). This maintains a stable chemical environment, allowing the rigorous identification of the specific constituent concentrations at the point of nucleation (Chayen, 1998 ▸), parameters that are typically obscured in vapor diffusion by the dynamic trajectory of equilibration. While protein concentration decreases following nucleation due to rapid solute depletion (McPherson & Gavira, 2014 ▸), the constant precipitant concentration (Baumgartner *et al.*, 2015 ▸) enables the precise mapping of phase boundaries from the solubility curve through the metastable zone. Despite these advantages, SX sample optimization remains heavily dependent on time-consuming, empirical adjustments to seeding protocols, incubation periods and crystallization conditions (Beale *et al.*, 2019 ▸; Stohrer *et al.*, 2021 ▸; Shoeman *et al.*, 2023 ▸). Consequently, systematic exploration of the phase space is essential to provide a rational guide for optimization.

Substantial effort has focused on tailoring microcrystal production, including mechanical fragmentation of macrocrystals (Dods *et al.*, 2017 ▸; Zielinski *et al.*, 2022 ▸; de la Cruz *et al.*, 2017 ▸), microseeding to enhance uniformity (Ibrahim *et al.*, 2015 ▸, Kupitz *et al.*, 2014 ▸), crystal screening and characterization methods (Darmanin *et al.*, 2016 ▸; Luft *et al.*, 2015 ▸), high-speed centrifugation to separate crystals by size (Tenboer *et al.*, 2014 ▸) and the establishment of condition-specific optimization protocols (Beale & Marsh, 2021 ▸; Stohrer *et al.*, 2021 ▸). Droplet microfluidics has also enabled high-throughput screening and crystallization, owing to its ability to generate highly monodisperse microcrystal suspensions (Stubbs *et al.*, 2024 ▸; Heymann *et al.*, 2014 ▸; Babnigg *et al.*, 2022 ▸). While valuable, these approaches remain predominantly rooted in trial and error. Critically, no current strategy combines the robust scalability and reproducibility of microbatch-under-oil crystallization with automated phase diagram generation, a gap that must be addressed to transition from empirical screening to the rational production of microcrystal suspensions suitable for SX.

Here, we report the development of an automated microbatch-under-oil crystallization approach for the rapid and low-volume (15–60 µl or ∼0.15–3.8 mg protein) determination of comprehensive phase diagrams. Our design employs a diagonal sampling strategy that varies the protein-to-precipitant ratio to sample paths parallel to the expected solubility curve (Luft & DeTitta, 1997 ▸; Luft *et al.*, 2007 ▸; Snell *et al.*, 2008 ▸). This targeted strategy identifies optimal conditions for controlled microcrystal nucleation and growth more efficiently than traditional orthogonal grids. To demonstrate a rational foundation for streamlining SX sample preparation, we applied this workflow to five protein targets selected to sample a diverse biophysical and crystallization space (Table 1 ▸). This selection includes the well characterized benchmarks and model systems concanavalin A, phycocyanin and xylanase, alongside two targets for time-resolved serial crystallography: *Arabidopsis thaliana* pyridoxal 5′-phosphate synthase subunit 1.3 (*At*Pdx1.3) and *Burkholderia pseudomallei* sedoheptulose-7-phosphate isomerase (*Bp*GmhA). By evaluating the workflow against this varied set, we demonstrate its utility for both standard structural studies and the more stringent requirements of time-resolved experiments.

## 2. Materials and methods

### 2.1. Microbatch-under-oil phase diagram setup

Crystallization was performed using an Oryx8 robot (Douglas Instruments) equipped with a four-channel dispensing tip. We developed a custom script and user interface (available via the *WaspRun* menu; Supplementary Fig. S1) to automate the design and execution of diagonal phase-space sampling. Droplets (2 µl final volume) were dispensed into untreated hydrophobic silver vapor batch plates (VB-SILVER, Douglas Instruments) covered with 100% paraffin oil (HR3-411, Hampton Research) [Supplementary Fig. S1(*a*)]. Each drop comprised protein, crystallization cocktail and, where applicable, seed stock, with water as a diluent. The seed stock and crystallization cocktail were either pre-mixed or added independently to define metastable boundaries. The script automates volume calculations based on user-defined corner concentrations, well count and final drop volume [Supplementary Fig. S1(*b*)]. Successful drops were translated to larger volumes by dispensing directly into PCR tubes (housed within an MUHWA PCR tube rack; MH-901134) to generate suspensions of microcrystals. To ensure homogeneity, the robot was programmed to pause after dispensing each tube, allowing manual vortexing and rapid mixing. Crystallization outcomes were monitored after 24 h using 3D digital (HRX-01, Hirox) or inverted trinocular microscopy (Motic BA310E) for *At*Pdx1.3 (Rodrigues *et al.*, 2017 ▸) and *Bp*GmhA (Harmer, 2010 ▸), respectively. Crystallization trials were conducted using the conditions detailed in Table 1 ▸. Comprehensive crystallization protocols for all proteins tested and detailed microbatch-under-oil seeding protocols are provided in the supplementary information.

## 3. Results and discussion

### 3.1. Phase diagrams reveal unconventional and complex crystallization landscapes

**Figure 1:**
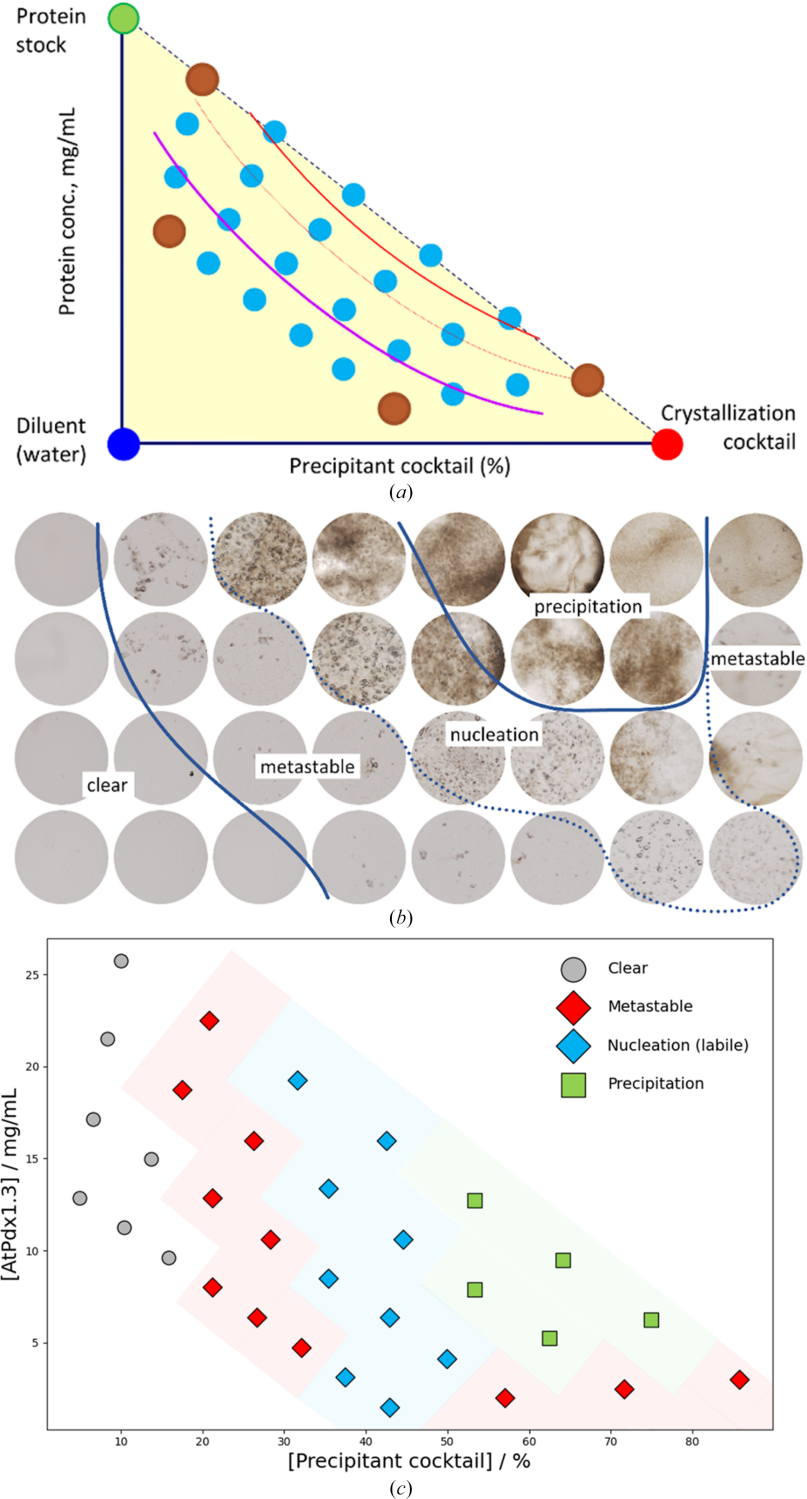
Automated microbatch-under-oil crystallization phase diagram of *At*Pdx1.3. (*a*) Schematic of the experimental design. Phase diagram points (yellow triangle) are generated by mixing a protein stock (green circle), crystallization cocktail (red circle) and diluent (blue circle) using a three-channel dispensing tip. Corner well concentrations (brown circles) are defined by the experimenter, with intermediate wells (cyan circles) interpolated by the software to create concentration gradients roughly parallel to the expected phase boundaries (red and purple lines). Drop volume and the number of wells are user-defined. The metastable boundary (purple line) is identified by repeating the experiment with seed stock, introduced via the crystallization cocktail or separately using a four-channel dispensing tip. (*b*) Representative 2.0 µl microbatch-under-oil drops in a typical *At*Pdx1.3 crystallization experiment. Crystals did not form in the metastable region unless seed stock was added. (*c*) Phase diagram derived from (*b*). Blue diamonds indicate nucleation-zone conditions without seed stock (spontaneous growth), while red diamonds indicate metastable conditions, where crystal growth required the addition of seed stock.

Proteins exhibit vast structural and functional diversity governed by unique physiochemical properties such as flexibility, oligomeric state, solubility and surface charges, which dictate their propensity to form a crystal (Chayen & Saridakis, 2008 ▸; McPherson & Gavira, 2014 ▸). While some globular proteins display classical phase diagrams, others present atypical, unpredictable phase boundaries, often overlooked by traditional sparse-matrix or grid-screening approaches (Beale *et al.*, 2019 ▸; Rupp, 2015 ▸). The automated microbatch-under-oil approach developed here addresses the mapping of phase boundaries by systematically sampling protein and precipitant concentrations across complete phase diagrams [Fig. 1 ▸(*a*)]. Using three stock components (protein, crystallization cocktail and diluent) dispensed by a liquid-handling robot (Supplementary Fig. S1), this method establishes concentration gradients that traverse the expected phase boundaries, ensuring comprehensive sampling of the metastable and nucleation zones. We applied this diagonal sampling strategy to several protein targets, focusing here on *A. thaliana* pyridoxal 5′-phosphate synthase subunit 1.3 (*At*Pdx1.3) and *B. pseudomallei* strain K96423 sedoheptulose-7-phosphate isomerase (*Bp*GmhA). Both are targets for time-resolved studies requiring precise crystal control to inform new mechanistic insights. While serial diffraction data for *At*Pdx1.3 (Stubbs *et al.*, 2024 ▸) and *Bp*GmhA (Tremlett *et al.*, in preparation) are addressed elsewhere, this study focuses specifically on the phase diagram methodology used to optimize these samples.

When applied to *At*Pdx1.3, the observed crystallization in the central nucleation zone was consistent with the known versatility of Pdx1 orthologs (Rodrigues *et al.*, 2017 ▸), whilst higher protein and precipitant concentrations led to precipitation throughout the drop [Fig. 1 ▸(*b*)]. While previous crystallization screens identified conditions that yielded crystals with high resolution and physical robustness for soaking experiments (Rodrigues *et al.*, 2017 ▸), our systematic mapping identified the precise boundaries that balance diffraction quality with the crystal size required for SX experiments [Fig. 1 ▸(*b*)]. Crucially, our approach unveiled two distinct metastable regions where crystal growth occurred exclusively upon the addition of seed stock (red diamonds) [Fig. 1 ▸(*c*)]. This dual metastable behavior is unconventional compared with model proteins such as concanavalin A, which exhibit classical, broad nucleation zones (Supplementary Fig. S2). We hypothesize that these separate metastable regions, one at high protein/low precipitant and another at low protein/high precipitant, reflect competing salting-in and salting-out effects. At lower salt concentrations salting-in is likely to increase solubility and suppress nucleation, whereas higher salt concentrations reduce solubility but nucleation remains kinetically hindered under oil, resulting in sparse crystal formation.

Mapping these distinct zones enables targeted optimization to meet specific SX requirements. For example, the low-precipitant metastable zone enhances sample efficiency by producing high-density suspensions with minimal reagents. Conversely, choosing between zones allows the management of experimental kinetics; while both yield microcrystals, higher precipitant concentrations increase the viscosity, potentially impeding ligand diffusion and kinetic synchronization in time-resolved mix-and-inject serial crystallography (MISC) experiments. Finally, moving precisely between nucleation and metastable regions via seeding decouples nucleation from growth, optimizing size and uniformity. This systematic approach transforms SX sample preparation from empirical trial and error into a rational strategy tailored to the physical demands of the diffraction experiment.

### 3.2. Seeded phase diagrams enable access to the crucial metastable zone

Unseeded phase diagrams rely on spontaneous nucleation and frequently contain regions where crystal growth is energetically favorable but nucleation is kinetically hindered. In contrast, microseeding introduces preformed (ideally homogeneous) crystal fragments (seeds) that serve as templates for crystal growth (Bergfors, 2003 ▸; Luft & DeTitta, 1999 ▸). By bypassing the stochastic and energetically demanding primary nucleation step, seeding promotes the oriented attachment of protein molecules directly onto existing lattices. This allows crystal production to be shifted from the nucleation zone into the metastable zone, providing a rational route to decouple nucleation from growth and achieve superior control over microcrystal size and density.

**Figure 2:**
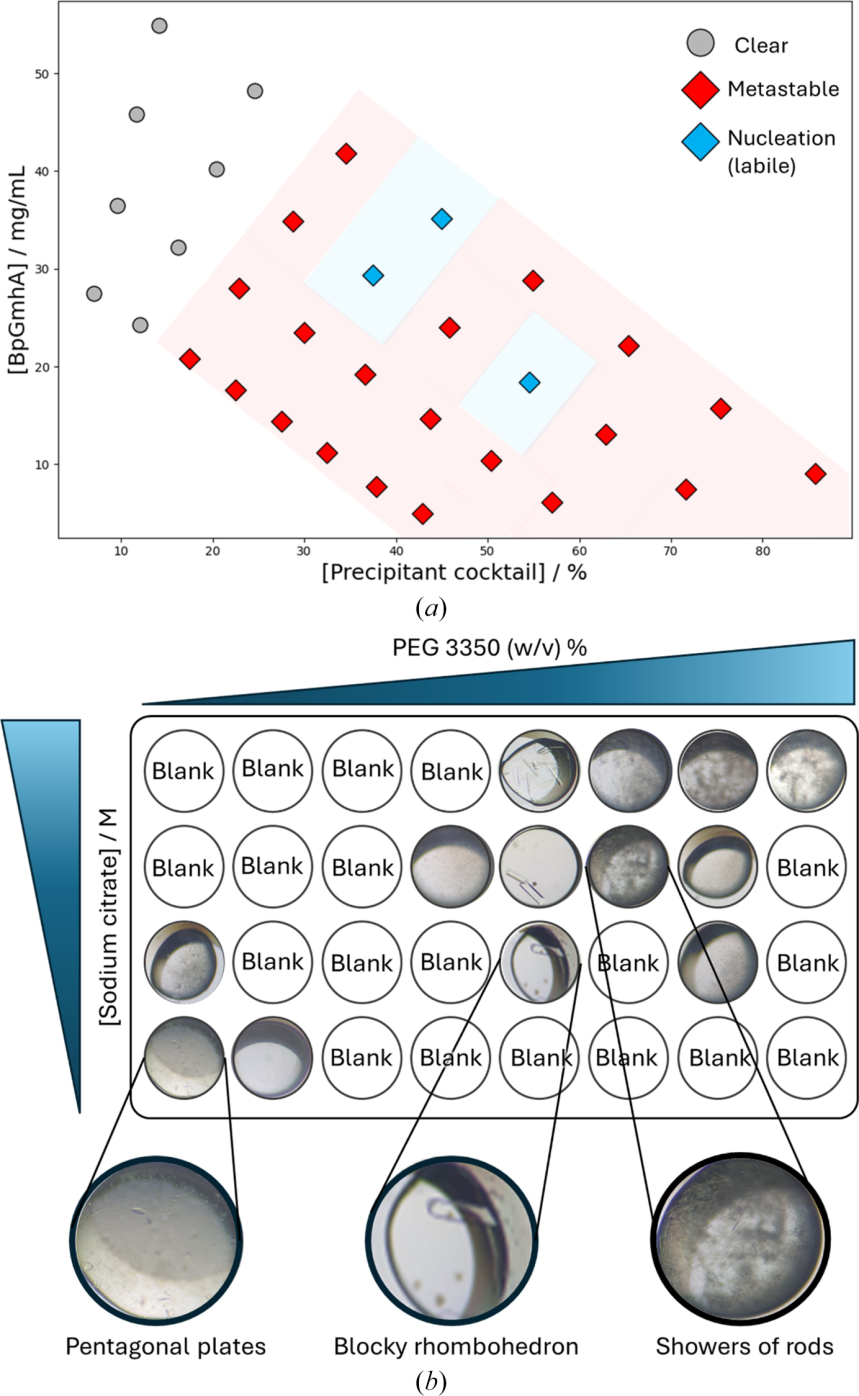
*Bp*GmhA microbatch-under-oil phase diagrams and morphology optimization. (*a*) Phase diagram of *Bp*GmhA comparing unseeded and seeded microbatch-under-oil crystallization experiments (see Supplementary Fig. S3). The data distinguish the nucleation zone (blue diamonds) where crystals formed without seeding and the metastable zone (red diamonds) where crystals form only upon seeding. Conditions yielding clear drops are indicated by gray circles. No precipitation phase was observed under the conditions tested. (*b*) The morphology of the *Bp*GmhA crystals varies across the precipitant–precipitant phase diagram. Three distinct crystal morphologies were observed by varying the concentrations of PEG 3350 and sodium citrate. Low concentrations of both precipitants yielded pentagonal plate-like crystals. At moderate-to-high sodium citrate and PEG 3350 concentrations, large rhombohedral crystals were obtained that were suitable for standard single-crystal experiments. Further increases in PEG 3350 concentration led to rod-shaped microcrystals that were more suitable for SX experiments.

For the five protein systems investigated in this study, microseeding expanded the accessible crystalline phase space by optimizing existing hits and providing access to previously unproductive metastable regions. Furthermore, the technique improved the morphological quality of the crystals. Growth with the metastable zone is characterized by lower supersaturation, which typically promotes a more orderly addition of molecules to the crystal lattice compared with the rapid growth associated with the labile zone (Bergfors, 2003 ▸; D’Arcy *et al.*, 2007 ▸). While visual morphology is not an absolute predictor of diffraction quality, the resulting crystals exhibited higher optical uniformity and facet definition, physical indicators of the controlled growth conditions often required to achieve high-resolution diffraction and low mosaicity in SX experiments. For *Bp*GmhA, seed stock was prepared from well ordered crystals (∼1.8 Å resolution in single-crystal cryocrystallography experiments; Tremlett *et al.*, in preparation), a strategy known to improve subsequent crystal order and resolution (Dods *et al.*, 2017 ▸). Upon seeding, conditions that previously yielded precipitates or remained clear produced crystals [Fig. 2 ▸(*a*)], indicating that these regions lie within or near the metastable zone. The seeds effectively shifted the system from a nucleation-limited environment to one favoring crystal growth, widening the accessible optimization landscape. Unseeded crystals exhibited ‘feather-like’ edges (Supplementary Fig. S3), a morphology associated with rapid growth and lattice defects driven by local supersaturation (Nanev, 2018 ▸; Yoshizaki *et al.*, 2002 ▸), resulting in no discernible diffraction. In contrast, seeded drops produced uniform, rod-shaped crystals with defined edges. This morphological transition typically reflects slower, more controlled crystal growth with uniform lattice ordering (Vekilov & Vorontsova, 2014 ▸) and, for the *Bp*GmhA system, correlates with the improved diffraction quality observed in SX experiments (Tremlett *et al.*, in preparation). By comparing these outcomes, the distinct thermodynamic and kinetic limitations of the crystallization landscape can be inferred.

**Figure 3:**
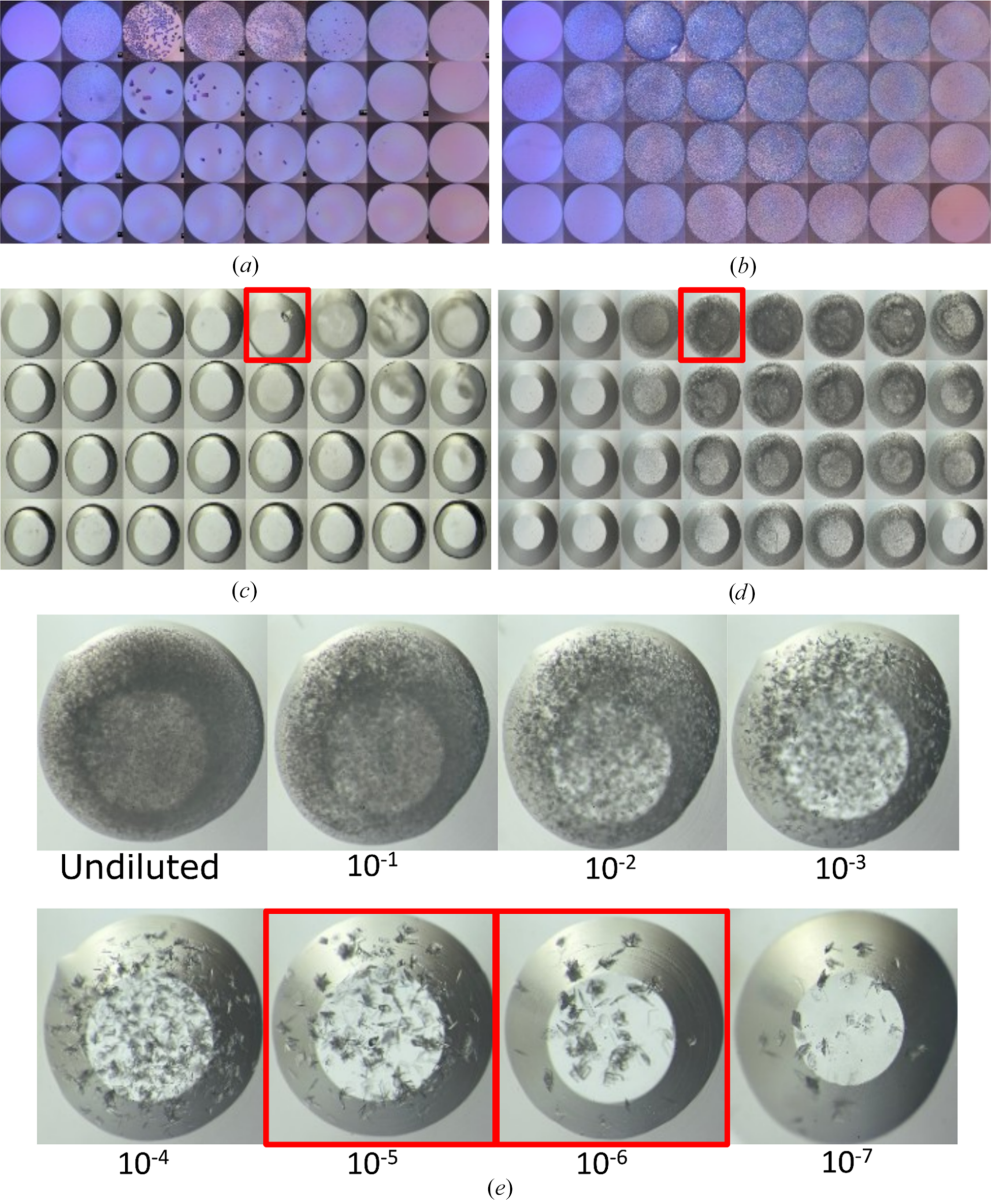
Automated microbatch-under-oil phase diagrams to rationalize serial crystallography sample preparation. (*a*, *b*) Phycocyanin phase diagrams obtained (*a*) without addition of seed stock and (*b*) with seed stock added. (*c*) Xylanase phase diagram obtained without the addition of seed stock, where the highlighted well (15.95 mg ml^−1^ xylanase in 4.46 *M* sodium formate) was used to generate seed stock for the subsequent xylanase phase diagram (*d*) with seed stock added. The highlighted well in (*d*) (20.1 mg ml^−1^ xylanase in 3.54 *M* sodium formate) was used for the xylanase seed-stock dilution series (*e*) using a cross-matrix experiment on the Oryx8 crystallization robot.

To further validate the methodology, we targeted phycocyanin (Doppler *et al.*, 2023 ▸) and xylanase, representing systems with distinct crystallization challenges. Phycocyanin was used to assess the transition from macrocrystal to microcrystal populations. The addition of seeds converted sparse clusters of large crystals into dense showers of uniform microcrystals across the majority of the phase diagram, significantly expanding the crystal-containing region [Figs. 3 ▸(*a*) and 3 ▸(*b*)]. Conversely, xylanase demonstrates the ability to rescue systems with extremely limited initial hits. In the unseeded phase diagram, only a single well yielded an observable crystal, defining a narrow nucleation zone [Fig. 3 ▸(*c*)]. This sole hit was used to generate seed stock, which subsequently unlocked a broad metastable region covering nearly 75% of the phase space with dense microcrystal showers [Fig. 3 ▸(*d*)]. This confirms that microseeding can effectively recover systems that are otherwise nucleation-limited (D’Arcy *et al.*, 2007 ▸; Obmolova *et al.*, 2014 ▸). Following this success, we performed a cross-matrix seed-stock titration experiment (from neat to 10^−7^ dilution) to optimize the seed-stock concentration [Fig. 3 ▸(*e*)]. Targeted phase diagrams using 10^−5^ and 10^−6^ dilutions revealed that outcomes can be precisely tailored. The 10^−5^ seed concentration consistently produced microcrystal clusters, while the 10^−6^ dilution yielded conditions with harvestable single crystals [Supplementary Figs. S4(*a*) and S4(*b*)]. Such titration allows the rational selection of conditions based on the experimental requirements, from dense suspensions of microcrystals for SX to larger crystals for preliminary diffraction testing.

### 3.3. Accessing crystal morphologies with microbatch-under-oil phase diagrams

Initial 96-well sparse-matrix screening of *Bp*GmhA identified sodium citrate and PEG 3350 as effective precipitants, consistently yielding large, well diffracting single crystals. These precipitants were selected for systematic phase-space mapping, where we deviated from the traditional protein versus precipitant phase diagram and instead simultaneously varied the concentrations of two precipitants. This approach generated a new phase diagram that successfully identified three distinct *Bp*GmhA crystal morphologies. Low precipitant concentrations favored small pentagonal plates, suggesting growth dominated by anisotropic surface kinetics and a step-flow mechanism, favoring two-dimensional expansion (Schmit & Dill, 2012 ▸; Nanev, 2018 ▸), while higher concentrations yielded large rhombohedral crystals and dense showers of rod-like microcrystals [Fig. 2 ▸(*b*)]. These high-concentration morphologies are indicative of diffusion-limited growth where the thermodynamic driving force for crystallization exceeds the rate of protein delivery to the crystal surface.

The rod and plate morphologies were prioritized for time-resolved MISC optimization and screened at the ESRF ID29 beamline (Orlans *et al.*, 2025 ▸). The plate-like crystals exhibited limited diffraction due to their thin dimensions and preferred orientation, whereas the rod-shaped microcrystals yielded complete data sets to ∼2.0 Å resolution (Tremlett *et al.*, in preparation). Consequently, the rod-shaped formulations were prioritized for scale-up. Similarly, an elongated diamond morphology observed for *At*Pdx1.3 (Supplementary Fig. S5) was excluded from further optimization after initial screening revealed no discernible diffraction. These results underscore the strategic value of integrating early small-volume SX diffraction screening using sample-efficient sheet-on-sheet (SOS) chips (Doak *et al.*, 2024 ▸) with phase diagram trials. This approach avoids the assumption that morphological perfection correlates with diffraction quality, ensuring that scale-up efforts are focused only on crystals that meet the resolution and stability requirements of the proposed experiment.

### 3.4. Scaling up microbatch-under-oil phase diagrams for serial crystallography

**Figure 4:**
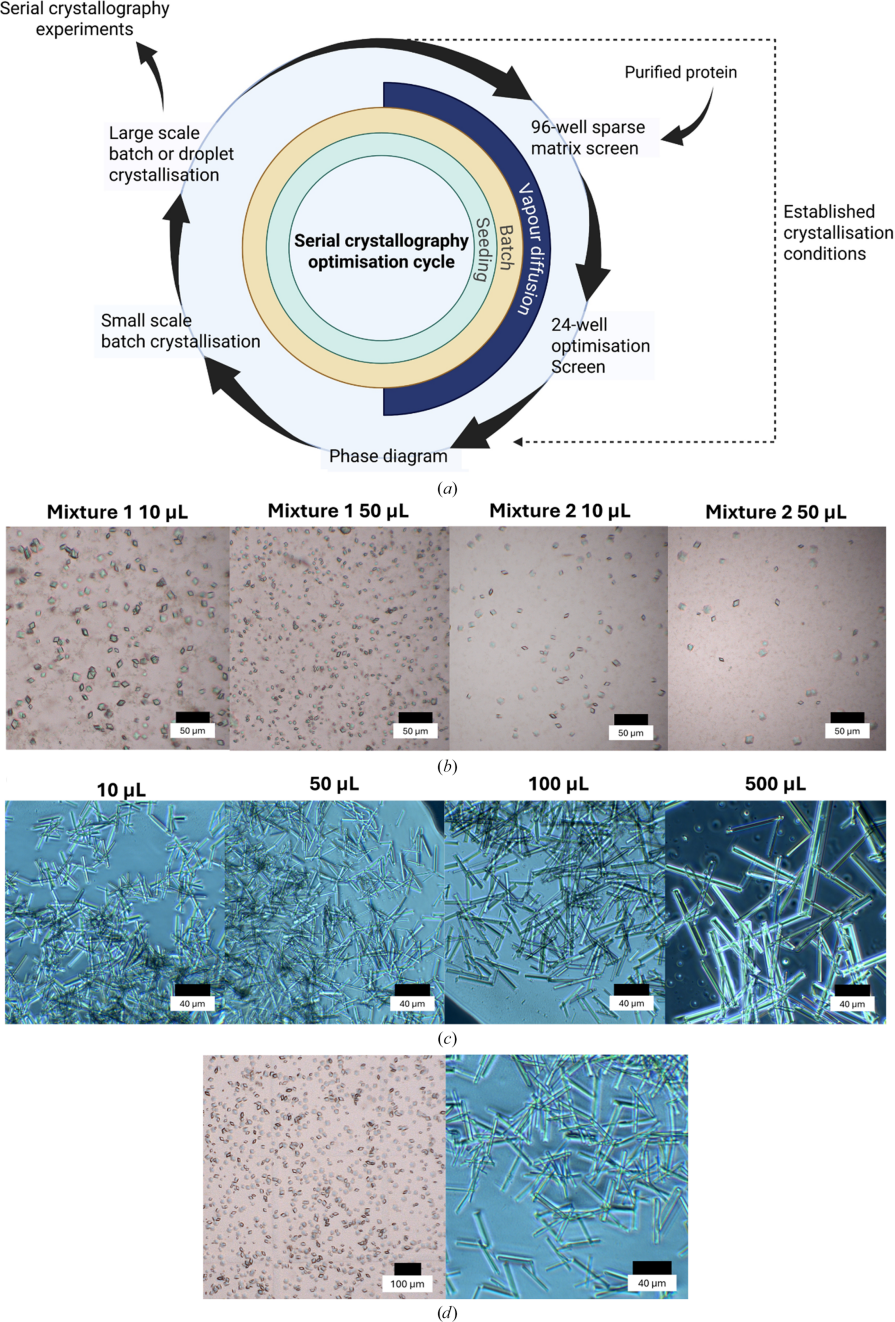
Optimization workflow for serial crystallography sample preparation. (*a*) Proposed workflow for microcrystal optimization, starting from purified protein through initial screening and detailed phase diagram analysis to identify crystallization boundaries. The optimal condition is then scaled up using batch or droplet methods into larger volumes suitable for SX experiments. (*b*) *At*Pdx1.3 crystals showed limited size changes when scaled from 10 µl to large volumes, indicating a volume-independent system at this scale. (*c*) Crystal size variation during scale-up of *Bp*GmhA. Initial scale-up from 10 µl to 500 µl resulted in rod-shaped crystals up to ∼200 µm, requiring crystal size reduction by doubling the seed concentration for SX experiments. (*d*) Final optimized *At*Pdx1.3 and *Bp*GmhA microcrystal suspensions suitable for SX experiments.

While microbatch-under-oil screening provides rapid insights, scaling to volumes suitable for SX is often hindered by altered nucleation kinetics and vessel geometry (Stohrer *et al.*, 2021 ▸; Beale *et al.*, 2019 ▸). Volume increases often exacerbate supersaturation gradients, leading to heterogeneous nucleation and growth, causing microscale conditions to fail (Beale *et al.*, 2019 ▸). To address this, we centered our optimization workflow on phase diagrams [Fig. 4 ▸(*a*)] to guide the transition from 2 µl drops to larger batch volumes. Validating conditions in small-scale batch trials using PCR tubes (10 µl) offers a risk-averse strategy to minimize sample consumption while transitioning away from paraffin oil [Supplementary Fig. S6(*a*)]. Automated dispensing and manual vortexing were critical to ensure thorough mixing [Supplementary Fig. S6(*b*)], preventing localized supersaturation gradients that could lead to heterogeneous nucleation (Mahon *et al.*, 2016 ▸).

For *At*Pdx1.3, seeded conditions corresponding to the nucleation zone yielded reproducible microcrystal batches (10^5^–10^6^ crystals per millilitre) at 10 µl volumes, whereas metastable zone conditions failed to produce sufficient crystals even with seed stock. Two successful formulations (Mixture 1, 13.3 mg ml^−1^*At*Pdx1.3 with 350 m*M* sodium citrate, 60 m*M* HEPES pH 7.0 and neat seed stock; Mixture 2, 6.735 mg ml^−1^*At*Pdx1.3 with 430 m*M* sodium citrate, 71 m*M* HEPES pH 7.0 and neat seed stock) produced ∼10–18 µm crystals [Fig. 4 ▸(*b*)]. When scaled to 50 µl, minor shifts in crystal size and density occurred, illustrating the sensitivity of the system to reaction volume [Fig. 4 ▸(*b*)]. Ultimately, a robust 150 µl seeded-batch preparation (a 1:1:1 ratio of 12 mg ml^−1^*At*Pdx1.3, 600 m*M* sodium citrate, 100 m*M* HEPES pH 7.0 and a 1:100 diluted seed stock) in Eppendorf tubes produced uniform 20 µm diamond-shaped crystals at 10^7^ crystals per millilitre [Fig. 4 ▸(*d*)]. These optimized microcrystal suspensions previously yielded 2.5 Å resolution serial data at ESRF ID29 (Stubbs *et al.*, 2024 ▸).

For *Bp*GmhA, the same workflow as above was implemented with a final formulation of 20 mg ml^−1^*Bp*GmhA, with 18%(*w*/*v*) PEG 3350, 250 m*M* sodium citrate pH 4.5 and a 1:200 diluted seed stock [Fig. 4 ▸(*d*)]. The seeded conditions were reproduced in PCR tubes up to 50 µl [Fig. 4 ▸(*c*)]. However, in larger 1.5 ml Eppendorf tubes (>100 µl) reduced nucleation and longer growth periods favored elongated crystals too large for capillaries, posing a risk of clogging and increased ligand-diffusion times (Tremlett *et al.*, 2025 ▸) during time-resolved MISC experiments [Fig. 4 ▸(*c*)]. To mitigate this, we utilized the phase diagram to re-optimize nucleation. By increasing the seed volume from 167 to 250 µl (producing a final 500 µl at a protein:precipitant:seed ratio of 1:1:2), we increased the available templates to outrun the growth of larger crystals, effectively depleting the protein pool to favor a dense population of smaller rods (∼35–45 µm) [Fig. 4 ▸(*d*)]. This iterative tuning of seed concentration ensures that target morphology and size is maintained across scales (D’Arcy *et al.*, 2014 ▸; Zhang *et al.*, 2021 ▸). Optimized *Bp*GmhA microcrystal suspensions are the focus of a separate manuscript currently in preparation (Tremlett *et al.*, in preparation).

Ultimately, the choice of scaling path is dictated by the sample-delivery strategy and the requirements of the proposed SX experiment (for a full discussion, see Table 1 in Tremlett *et al.*, 2025 ▸). Time-resolved studies often necessitate larger volumes of highly concentrated and uniform microcrystal suspensions to ensure kinetic synchronization across timepoints. While large volume suspensions are typically required for more demanding liquid jets, sample-efficient platforms such as SOS chips (Doak *et al.*, 2024 ▸) offer a robust alternative for static serial experiments, with sample volumes as low as ∼3–5 µl. These platforms mitigate the risk associated with volume scaling by allowing the use of smaller, more predictable batches, which were previously validated during initial phase diagram mapping. This strategic alignment between phase diagram guidance and experimental design ensures that sample preparation is optimized for the specific kinetic and physical demands of the diffraction experiment.

## 4. Conclusion

Phase diagrams remain a critical yet under-utilized framework for rational crystallization optimization. The automated microbatch-under-oil crystallization approach presented here transforms phase diagram mapping from a sample-intensive hurdle into a high-throughput tool. By utilizing a novel diagonal sampling strategy and as little as 15–60 µl of protein, we successfully identified complex landscapes that are typically invisible to standard sparse-matrix screens. Crucially, the integration of microseeding and seed-stock titration enables the predictive rescue of nucleation-limited systems. This systematic decoupling of nucleation from growth provides a robust mechanism to prioritize specific crystal morphologies optimized for serial crystallography, ensuring that scale-up to larger batches remains reproducible by effectively outrunning macrocrystal formation. Ultimately, centering crystallization workflows on seeded microbatch-under-oil phase diagrams provides a robust, rational strategy for serial crystallography sample preparation.

## 5. Related literature

The following references are cited in the supporting information for this article: Chayen (1998 ▸), Doppler* et al.* (2023 ▸), Luft & DeTitta (1999 ▸), Shaw Stewart & Conti (1995 ▸),Tremlett* et al.* (2025 ▸).

## Supplementary Material

Materials and methods, and supplementary figures. DOI: 10.1107/S2052252526000448/rs5008sup1.pdf

## Figures and Tables

**Table 1:** Proteins used in this study

Protein	Monomer molecular weight (kDa)	Stock concentration (mg ml^−1^)	Protein buffer	Crystallization condition
*Arabidopsis thaliana* pyridoxal 5′-phosphate synthase subunit 1.3 (*At*Pdx1.3)	34.2	30	200 m*M* KCl, 20 m*M* Tris pH 8.0	1 *M* sodium citrate, 167 m*M* HEPES pH 7.0
*Burkholderia pseudomallei* strain K96423 sedoheptulose-7-phosphate isomerase (*Bp*GmhA)	21.5	64	20 m*M* HEPES–NaOH, 200 m*M* NaCl pH 7.5	250 m*M* sodium citrate pH 4.5, 40%(*w*/*v*) PEG 3350
Concanavalin A from *Canavalia ensiformis*	26.5	10	MQ water	4 *M* ammonium sulfate
Phycocyanin from *Thermosynechococcus elongatus*	37.4	50	30 m*M* HEPES pH 7.0, 75 m*M* MgCl_2_	75 m*M* HEPES pH 7.0, 20 m*M* MgCl_2_, 45%(*w*/*v*) PEG 3350
Xylanase	22	36	0.2 *M* sodium phosphate pH 7.0, 43%(*v*/*v*) glycerol	8.0 *M* sodium formate
