# Supplementary material for: Automated microbatch-under-oil phase diagrams to rationalize serial crystallography sample preparation
Source: IUCrJ. 2026 Jan 27;13(Pt 2):159–68. doi: 10.1107/S2052252526000448 (PMC12951833; doi:10.1107/S2052252526000448)
Supplement: Supplementary file 1 [file m-13-00159-sup1.pdf]

IUCrJ

Volume 13 (2026)

Supporting information for article:

Automated microbatch-under-oil phase diagrams to rationalise serial crystallography sample preparation

Jack Stubbs, Courtney J. Tremlett, Abigail Waitman, Nicholas J. Harmer, Allen M. Orville, Ivo Tews, Stefan Kolek and Patrick D. Shaw Stewart

S1. Materials and Methods

S1.1 *At*Pdx1.3 expression, purification and crystallization

Pyridoxal 5'-phosphate synthase subunit 1.3 (Pdx1.3) from *Arabidopsis thaliana* (UniProt ID: Q8L940; EC: 4.3.3.6) was expressed in *Escherichia coli* BL21 (DE3) cells at 37°C in Luria-Bertani (LB) broth supplemented with 100 µg/mL ampicillin. Protein expression was induced by adding 25% (w/v) lactose and incubating for 16 hours at 30°C after the cultures reached an OD_{600} of 0.6 - 0.8. Cells were harvested by centrifugation at 6240 x *g* for 30 minutes at 4°C and stored at -80°C. Thawed cell pellets were resuspended in 20 mL lysis buffer (50 mM Tris (pH 7.5), 500 mM sodium chloride, 10 mM imidazole, 2% (v/v) glycerol) and lysed via sonication on ice for 3 min at 50% amplitude. Following clarification by centrifugation at 140 000 x *g* for 1h at 4°C, the supernatant was filtered (0.45 µm) and applied to a 1 mL HisTrap HP column (GE Healthcare) for metal ion affinity chromatography. *At*Pdx1.3 was washed and eluted with lysis buffer containing 50 mM and 500 mM imidazole, respectively, both supplemented with 5% (v/v) glycerol.

Eluted protein was desalted and buffer-exchanged into gel filtration buffer (20 mM Tris (pH 8.0), 200 mM KCl) using a PD-10 column. Fractions containing pure *At*Pdx1.3 (verified by A_{280} and SDS-PAGE) were pooled and concentrated to 30 mg/mL using a 30 kDa Vivaspin 20 centrifugal concentrator (Sartorius). To remove aggregates or amorphous material, the concentrated protein was centrifuged at 10 000 x g for 30 minutes at 4°C prior to crystallization. Seed stock was prepared by growing crystals overnight from a 10 µL vapor diffusion setup (1:1 ratio of ∼12 mg/mL AtPdx1.3 to mother liquor: 600 mM sodium citrate, 100 mM HEPES (pH 7.0)) in 24-well XRL plates (Molecular Dimensions). Crystals were harvested and dispersed by vortexing with a Hampton Seed Bead for 3 minutes, alternating between 30 seconds of vortexing and 30 seconds on ice, following the seed bead method (Luft & DeTitta, 1999); the resulting seed stock was stored at −20°C.

S1.2 *Bp*GmhA expression, purification and crystallization

Sedoheptulose-7-phosphate isomerase (GmhA) from *B.pseudomallei* strain K96423 (UniProt ID: Q93UJ2; EC: 5.3.1.28) was expressed in *Escherichia coli* BL21 (DE3) cells (Merck #69450-3) in Luria-Bertani (LB) broth supplemented with 100 µg/mL kanamycin. Cells were initially incubated at 37°C and 220 RPM until OD_{600} reached 0.6, after which expression was induced using ZYM-5052 media at 20°C for 19 hours. Cells were harvested by centrifugation at 4500 x *g* for 30 minutes at 4°C and stored at -80°C. Thawed cell pellets were resuspended in a lysis buffer (20 mM Tris-HCl, (pH 8.0) and 500 mM NaCl). Cells were lysed using an Ultrasonics VCX-130 at 70 % amplitude for 6 minutes using 20 seconds on and 20 seconds off pulses over ice. The lysate was clarified by centrifugation at 24,000 x *g* for 30 minutes at 4°C, and the resulting supernatant was filtered (0.22 µm). The clarified lysate was purified via gravity flow using 6 mL of nickel resin beads (Cytiva #10249123). Beads were first washed with 20 mM Tris-HCl (pH 8.0), 500 mM NaCl and 20 mM

imidazole, and *Bp*GmhA was then eluted using 20 mM Tris-HCl (pH 8.0), 500 mM NaCl and 500 mM imidazole. Eluted protein was then loaded onto a Superdex 200 16/600 HR column (Cytiva #28989335) using an Äkta Pure 25 L (Cytiva #29018224) and eluted isocratically in 20 mM HEPES-NaOH, 200 mM NaCl (pH 7.5). TEV protease was added to GmhA at 1:100 dilution and incubated for 36 hours at 4°C. The sample was repurified by nickel affinity chromatography to collect the untagged protein and was then passed through the size exclusion column again as above. *Bp*GmhA was finally concentrated to 64 mg/mL using a 30 kDa Amicon Ultra 15 mL centrifuge device (Merck, #UFC903008).

Seed stock was prepared by mixing 10 mg/mL GmhA with mother liquor (100 mM sodium citrate, 21 % (w/v) PEG 3350, 9 % DMSO (pH 4.5)) in a 1:1 ratio. This mixing occurred in microbatch-under-oil plates using an Oryx6 crystallization robot (Douglas Instruments), and plates were incubated at 20°C. All formed crystals were collected and dispersed by sonication on ice for 70 seconds at 80 % amplitude. The resulting seed stock was stored at −20°C.

S1.3 Concanavalin A crystallization

Concanavalin A from *Canavalia ensiformis* (jack bean) was obtained in powdered form from Sigma-Aldrich (L7647). Crystallization of concanavalin A was performed using a protein stock at 10 mg/mL. The crystallization cocktail comprised 4 M ammonium sulfate.

S1.4 Phycocyanin crystallization

Crystallization of phycocyanin from *Thermosynechococcus elongatus* was performed using a protein stock at 50 mg/mL. The crystallization cocktail comprised 75 mM HEPES (pH 7.0), 20 mM $MgCl_2$, and 45% (w/v) PEG 3350. Phycocyanin protein and seed stock were kindly provided by Jui-Tung Tseng and Dr. Jay-How Yang (Arizona State University). Purification and crystallization protocols are detailed in Doppler *et al.*, 2023.

S1.5 Xylanase crystallization

Xylanase was purchased as a 36 mg/mL solution in 0.2 M sodium phosphate (pH 7.0), 43% (v/v) glycerol (Macro Crystal Oy). Crystallization of xylanase was performed using a protein stock at 36 mg/mL, a crystallization cocktail comprising 8.0 M sodium formate, and water as a diluent. Seeds were prepared from a crystallization hit identified in the original microbatch-under-oil phase diagram experiment without seeding (15.95 mg/mL xylanase in 4.46 M sodium formate). Seed stock titration was performed using the cross-matrix experiment on the Oryx8 crystallization robot (Douglas Instruments), which systematically identifies appropriate seed dilutions for 20.1 mg/mL Xylanase in 3.54 M sodium formate. The microbatch-under-oil phase diagram was subsequently repeated with

diluted seed stock (10^{-5} and 10^{-6}), enabling reproducible identification of the nucleation zone and improved microcrystal production.

S1.6 Microbatch-under-oil seed stock preparation and crystal harvesting

Recognising that seed preparation methods and their specific execution vary widely across crystallization systems and research groups (Tremlett *et al.*, 2025), the following protocol outlines the standard procedure for preparing seed stock and harvesting crystals, which involves only slight adjustments to conventional techniques to accommodate the microbatch-under-oil environment. These methods have been previously published and described in detail (Chayen *et al.*, 1998, Shaw Stewart & Conti, 1995) but are highlighted and summarised below.

The process for preparing a seed stock is initiated by working directly within the well. This involves gently wicking or pouring away excess oil followed by crushing the crystal material in the remaining drop using a fine probe. Next approximately 2 µL of the original hit solution is pipetted onto the crushed material; using small tips (gel loading tips work well) is recommended to prevent aspirating unwanted oil. The resulting mixture is homogenized, transferred to a seed bead tube, and subsequently vortexed vigorously for 4 x 30 seconds on ice. While minor amounts of included oil are acceptable, the final stock must always be stirred or vortex briefly before use, with aspiration taking place from the middle of the suspension to avoid floating oil. Although this is the standard approach, in the example of *Bp*GmhA, it was previously shown that seeds created using a seed bead were variable in size (Tremlett *et al*., 2025). To obtain homogeneous seeds, sonication was utilised (see supplementary S1.2). This resulted in more homogeneous seeds and crystals as a result, important for time-resolved mix-and-inject serial crystallography experiments (Tremlett *et al*., 2025). Therefore, it is again crucial to optimise each stage of crystallization to obtain reproducible crystals for the proposed experiment.

Crystal harvesting from the viscous oil environment presents a mechanical challenge, though reliable protocols exist. The presence of the oil layer, however, protects the crystals from physical shock, making unmounted crystals easily transportable. If crystals adhere to the supporting surface, they must be gently loosened (inside the drop) with microtools or a whisker. For standard transfer, a harvest solution must be added to the crystals prior to removal from the oil, requiring 15 - 30 µL of stabilising solution. Critically, this solution contains a slightly higher (~5%) concentration of precipitant than the drop. After a short equilibration period (up to 30 minutes), a pipette is used to withdraw the enlarged drop and crystal harvesting can be performed.

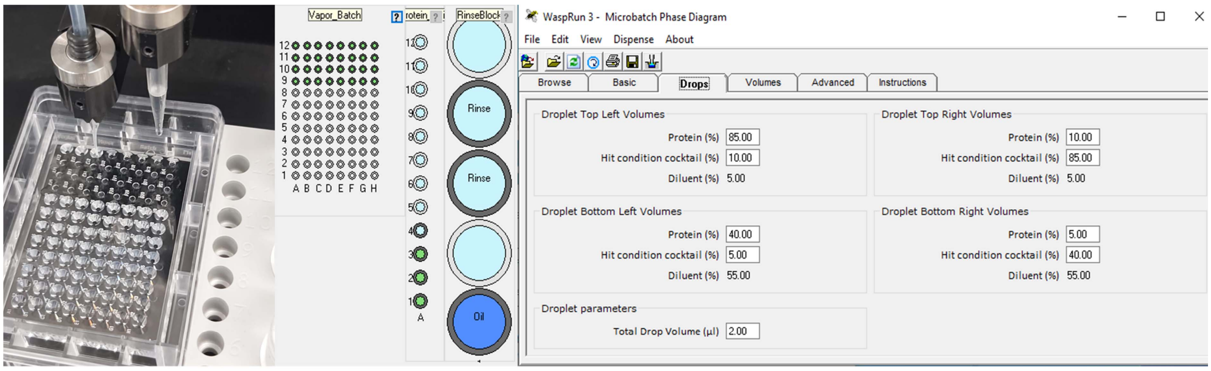

(a) (b)

Figure S1 Microbatch-under-oil experimental setup and automated dispensing script. (a) The protein stock, crystallization cocktail and diluent are simultaneously dispensed by a three or four channel dispensing tip into the microbatch plate to form an aqueous drop, which is immediately overlaid with 100% paraffin oil using the plastic V tip. (b) The experimenter selects the four corner wells of the phase diagram, after which the software automatically interpolates the remaining wells to generate an 8 x 4 array. Droplet volume, ratio of protein stock:crystallization cocktail, and the number of wells to be dispensed can be adjusted within the software menu prior to starting the experiment.

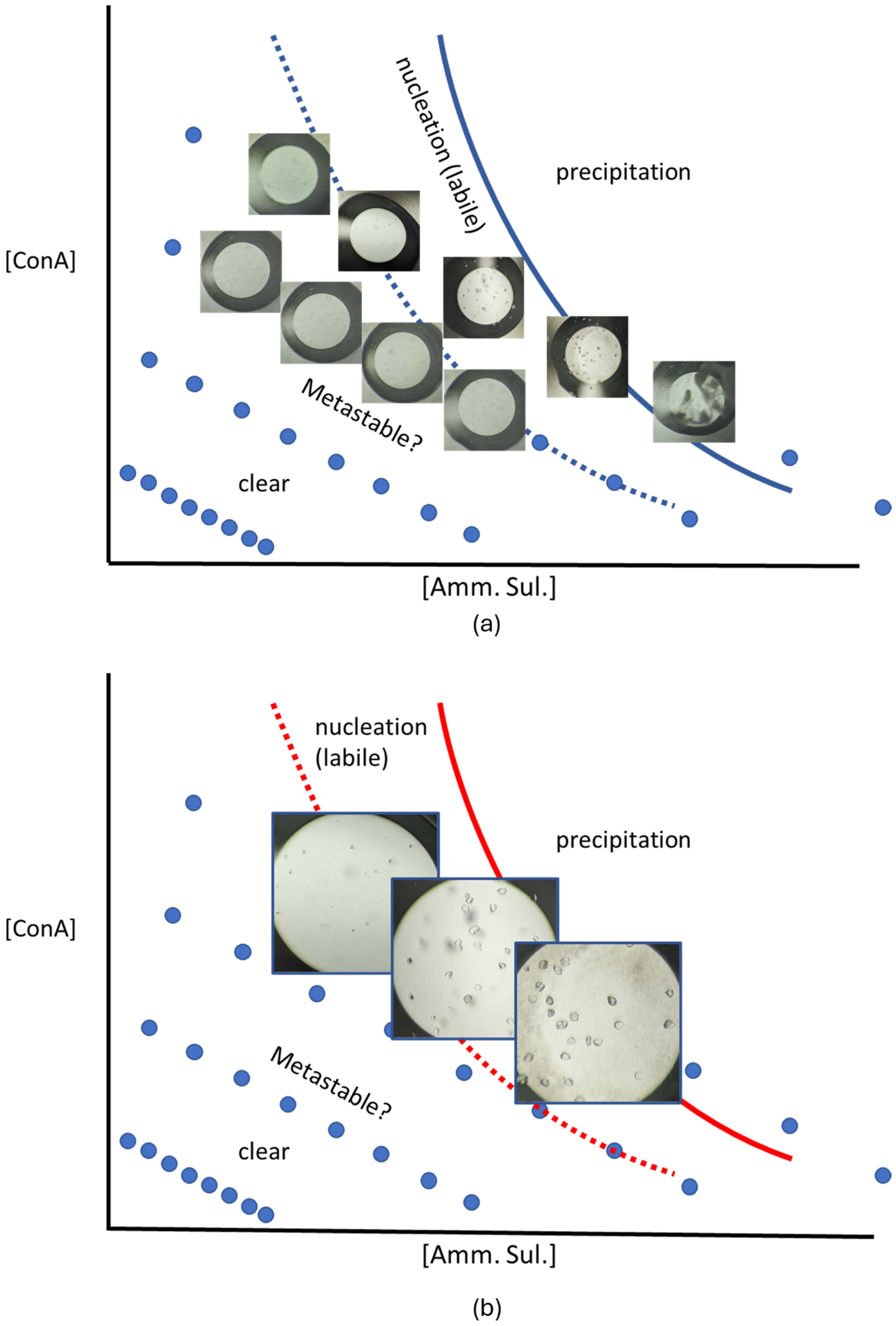

Figure S2 Concanavalin A microbatch-under-oil phase diagram. (a) Phase diagram obtained with an ammonium sulfate crystallization cocktail, without seed stock. (b) Crystals were observed in only three drops, defining the upper and lower boundaries of the nucleation zone.

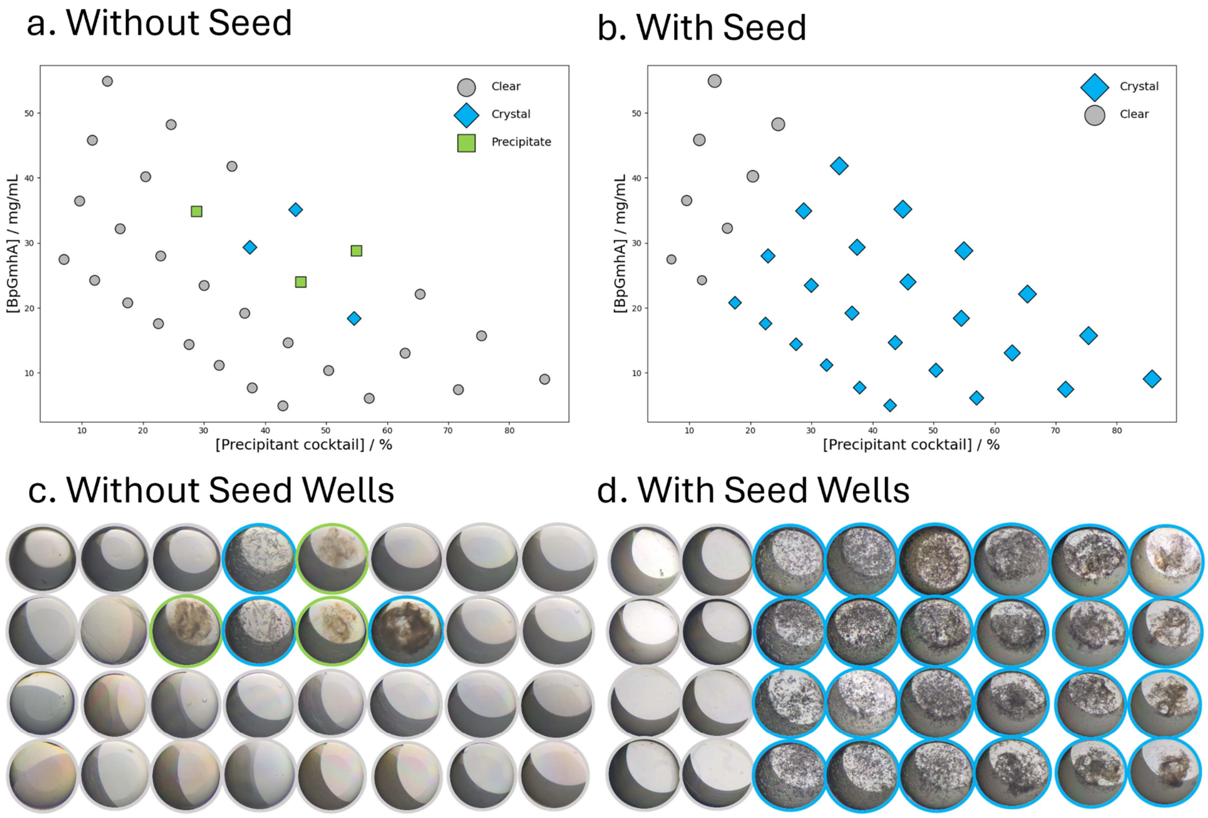

Figure S3 *Bp*GmhA microbatch-under-oil phase diagrams comparing unseeded and seeded crystallization outcomes. (a) Phase diagram obtained without addition of seed stock. The data points are plotted using the following key: grey circle for a clear drop, green box for precipitate, and blue diamond for crystal. (b) Phase diagram obtained with the addition of seed stock. The data points follow the same key as (a). (c) (d) Representative optical microscopy images from the unseeded and seeded phase diagrams, respectively.

(a) (b)

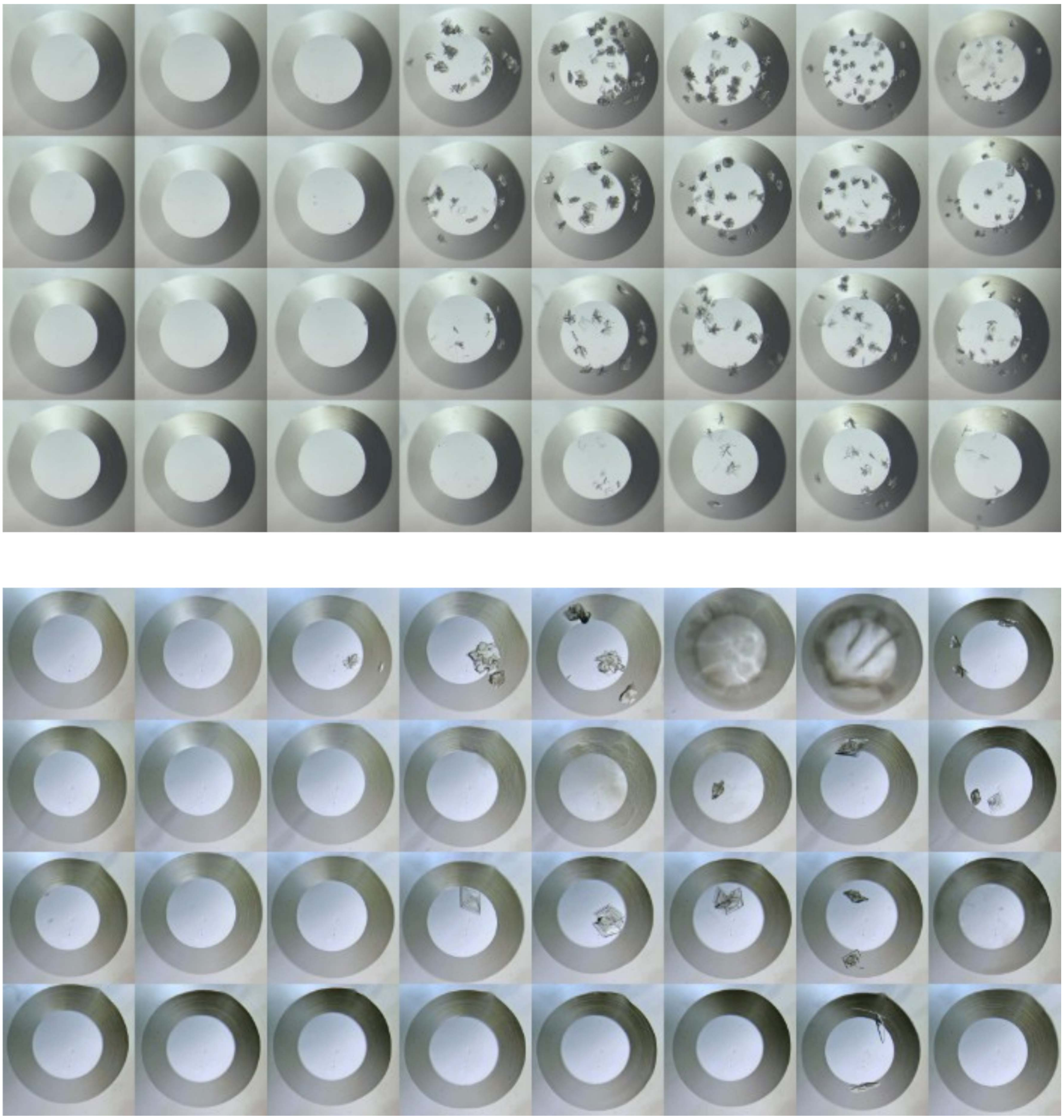

Figure S4 Effect of seed stock dilution on Xylanase microbatch-under-oil phase diagrams. (a) Seeded microbatch-under-oil phase diagram with 10^{-5} diluted seed stock generated in Fig 3(e). (b) Seeded microbatch-under-oil phase diagram with 10^{-6} diluted seed stock generated in Fig 3(e).

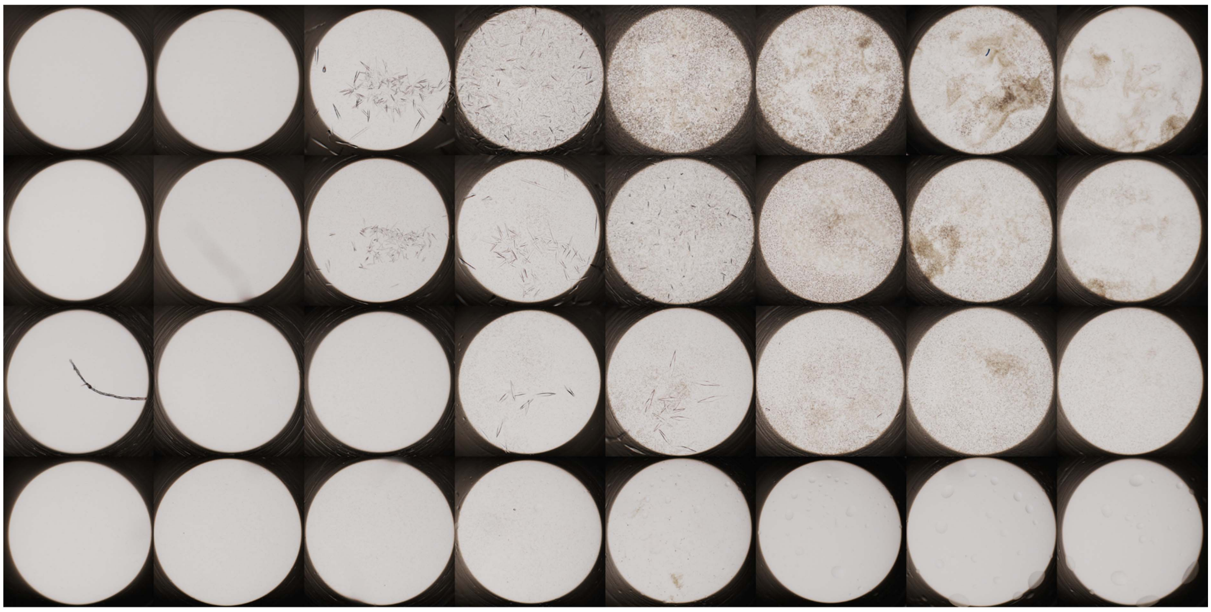

(a)

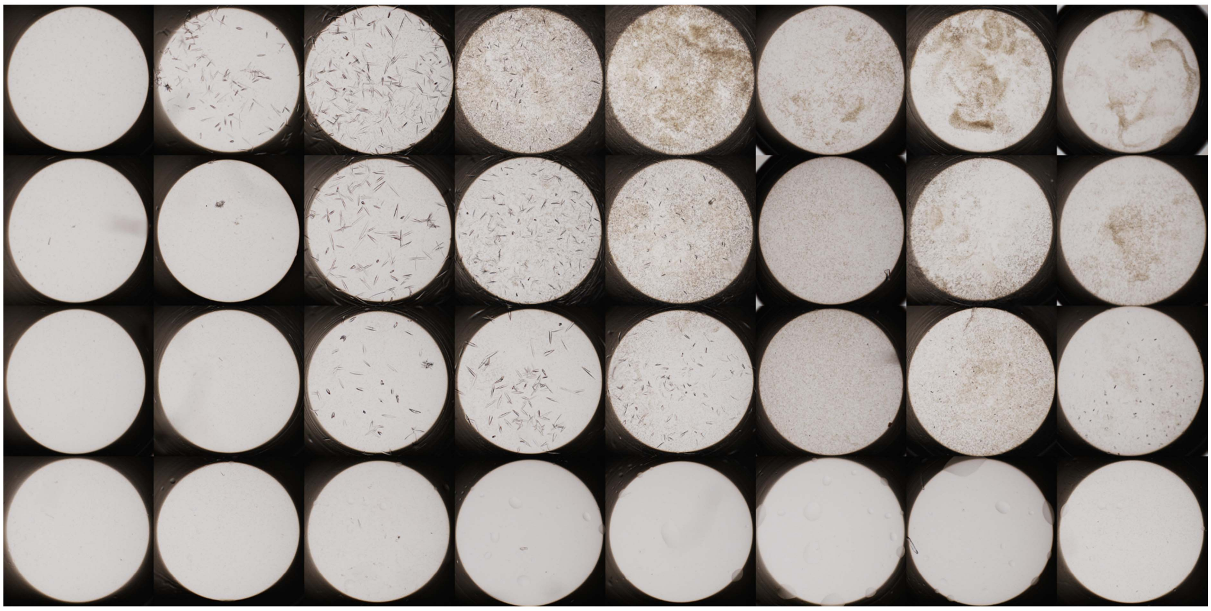

(b)

Figure S5 Microbatch-under-oil phase diagrams for *At*Pdx1.3 crystallization using an alternative crystallization cocktail. The crystallization cocktail consisted of 15% (w/v) PEG 8000 and 250 mM sodium cacodylate (pH 6.5). (a) Phase diagram obtained without addition of seed stock. (b) Phase diagram obtained with the addition of seed stock (as described in S1.1).

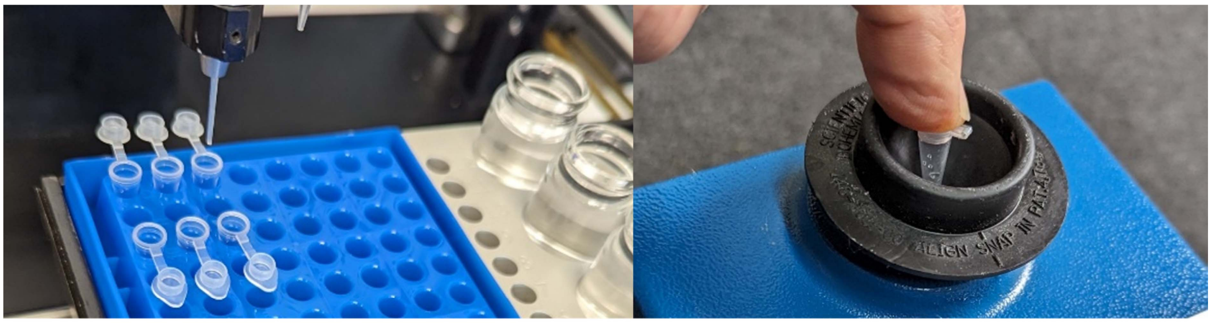

(a) (b)

Figure S6 Scaling up using the microbatch-under-oil script without paraffin oil. (a) Replacement of the microbatch plate with a 96-well PCR tube rack enables precise dispensing of previously successful crystallization conditions, with the option to increase sample volumes. (b) A programmed pause allows manual vortexing of the PCR tube to ensure thorough mixing of the crystallization mixture prior to incubation.

References

Chayen, N. E. (1998). *Acta Cryst D* **54**, 8–15.

Doppler, D., Sonker, M., Egatz-Gomez, A., Grieco, A., Zaare, S., Jernigan, R., Domingo Meza-Aguilar, J., T. Rabbani, M., Manna, A., C. Alvarez, R., Karpos, K., Villarreal, J. C., Nelson, G., Yang, J.-H., Carrion, J., Morin, K., K. Ketawala, G., L. Pey, A., Angel Ruiz-Fresneda, M., Luis Pacheco-Garcia, J., A. Hermoso, J., Nazari, R., Sierra, R., S. Hunter, M., Batyuk, A., J. Kupitz, C., E. Sublett, R., Lisova, S., Mariani, V., Boutet, S., Fromme, R., D. Grant, T., Botha, S., Fromme, P., A. Kirian, R., Manuel Martin-Garcia, J. & Ros, A. (2023). *Lab on a Chip* **23**, 3016–3033.

Luft, J. R. & DeTitta, G. T. (1999). *Acta Cryst D* **55**, 988–993.

Shaw Stewart, P. D. & Conti, E. (1995). Douglas Instruments Research Report 3, http://www.douglas.co.uk/rep3.htm.

Tremlett, C. J., Stubbs, J., Stuart, W. S., Shaw Stewart, P. D., West, J., Orville, A. M., Tews, I. & Harmer, N. J. (2025). *IUCrJ* **12**, 262–279.
